# The intensification of the strongest nor’easters

**DOI:** 10.1073/pnas.2510029122

**Published:** 2025-07-14

**Authors:** Kevin Chen, Xueke Li, Mackenzie M. Weaver, Shannon A. Christiansen, Annabelle L. Horton, Michael E. Mann

**Affiliations:** ^a^Department of Physics and Astronomy, University of Pennsylvania, Philadelphia, PA 19104; ^b^Department of Earth and Environmental Science, University of Pennsylvania, Philadelphia, PA 19104

**Keywords:** nor’easters, extratropical cyclones, climate change, intensity, precipitation

## Abstract

Nor’easters are coastal extratropical cyclones that feed upon both thermal contrasts (meridional and land-ocean) and oceanic latent heat release, causing them to intensify along the U.S. East Coast. With central pressures that sometimes rival those of tropical cyclones, they represent a significant coastal hazard and are often associated with strong winds, heavy snowfall, disruption, and damage. While interest in studying the impacts of climate change on storm behavior is growing, nor’easters have historically received far less attention than tropical cyclones, largely due to challenges in documenting and categorizing these storms combined with the relatively short observational record. Here, we address these challenges by employing a cyclone tracking approach in concurrence with long-term reanalysis data to create a reliable historical database of these storms. We find a significant increasing trend in the maximum wind speeds of the most intense (>66th percentile) nor’easters. We also observe an increasing trend in hourly precipitation rates associated with these storms. Such changes have profound implications for coastal cities and shorelines, increasing the risk of coastal flooding and erosion.

## Significance

Nor’easters, with their heavy precipitation and strong winds, pose significant threats to cities along the U.S. East Coast, often leading to devastating impacts. Some of the most notable nor’easters include the “Perfect Storm,” “Storm of the Century,” “Snowmaggedon,” and the January 2018 blizzard. Using a cyclone tracking approach combined with long-term reanalysis data, we present a comprehensive and homogeneous historical record of Atlantic nor’easters. Our analysis of nor’easter characteristics reveals that the strongest nor’easters are becoming stronger, with both the maximum wind speeds of the most intense (>66th percentile) nor’easters and hourly precipitation rates increasing since 1940, suggesting an additional contribution to coastal risk in a warming world.

Extratropical cyclones (ETCs)—low-pressure systems that form in the midlatitudes—are an important component of Earth’s atmospheric circulation, transporting moisture and energy and significantly influencing midlatitude weather and climate (1–6). ETCs are often accompanied by heavy precipitation and strong winds, contributing to over 70% of the precipitation in many regions of North America and Europe (7), as well as severe storm surge, coastal flooding, and sometimes massive blizzards (8, 9), which threaten human lives and critical infrastructures. For example, ETC Kyrill in 2007 incurred a cost of 7 billion Euros in damages and resulted in at least 46 deaths (10).

There is a general consensus that there will be fewer ETCs in a warmer climate, a robust trend seen in both historical records and climate models (4, 11–14). This decline is in part due to polar amplification of warming, wherein the polar regions warm more than lower latitude regions (15, 16). Polar amplification reduces the pole-equator temperature gradient, in turn reducing the baroclinic energy available for ETC formation (4). In the subtropics, increased atmospheric stability due to enhanced upper tropospheric warming also contributes to a reduction in cyclone activity (12).

While there is a consensus that there will be fewer ETCs in a warmer world, there is less consensus regarding changes in intensity. Current generation models fail to capture some relevant processes. For example, models tend to produce fewer, weaker, and slower-moving ETCs compared to reanalyses (17) due to a shallower dry air layer aloft and a less tilted vertical structure in the frontal region, caused by weaker ageostrophic circulation (1). Shifts in the position of the polar or subtropical jet stream, influenced by Arctic amplification and tropical heating, may give rise to changes in the complex interactions between jet stream perturbations and coastal low-level baroclinicity, impacting both the tracking and intensity of ETCs in ways that are challenging for models to accurately capture (2, 12, 18). Previous work has used varying metrics for measuring ETC intensity, such as minimum lifetime sea level pressure (SLP), maximum relative vorticity, and maximum sustained wind speeds, as well as varying classifications of cyclone strength, either based on wind speeds or precipitation intensity (19). In addition, limitations due to coarse model resolution can affect cyclone detection and tracking schemes (20, 21), with lower-resolution data generally leading to an underestimation of the number of detected ETCs (22, 23). There is, as a result of these confounding factors, considerable divergence in future projections of ETC intensity in past studies, with findings ranging from no significant change in median cyclone intensity (11), to a decrease (4, 24) or an increase (25, 26).

Most past studies, in addition, fail to adequately examine an important subclass of ETCs—coastal North Atlantic storms known as “nor’easters” (for the unusual northeasterly prevailing direction of the strongest winds), whose characteristics differ substantially from other ETCs. Commonly observed along the U.S. East Coast between September and April, these ETCs are influenced not just by meridional baroclinicity in the vicinity of the polar front, but by sharp zonal baroclinicity at the land/ocean boundary and latent heat release over the ocean. They are known to form through at least two distinct mechanisms: Miller type A cyclogenesis (27), driven by baroclinic instability (1, 4, 5, 8, 11) due to sharp land/ocean temperature gradients, or the Miller type B mechanism, which involves secondary cyclogenesis over coastal waters driven substantially by latent heat release (12, 28). Notable nor’easters include the “Perfect Storm” of 1991 (29), the “Storm of the Century” in 1993 (30), the “Snowmaggedon” storm of 2010 (31), and the January 2018 blizzard (32). Nor’easters have particularly severe and widespread societal and economic impacts because they pass over densely populated regions such as the Northeast corridor (8, 11, 12). Given such consequences, it is crucial to understand how nor’easters are changing in response to anthropogenic climate change.

While projected changes in overall ETC intensity remain unclear (4, 11, 24–26), several past studies argue for a potential intensification of nor’easters due to human-caused climate change (4, 8, 12, 20). Sharper land/ocean surface temperature contrasts and enhanced latent heating owing to rising ocean surface temperatures both lead to an intensification of storms. The enhanced moisture convergence due to increasing intensity, moreover, leads to increased precipitation rates (and further increases in intensity due to the added latent heating).

Heretofore, there has been little consensus on whether such trends are evident in the observational record. Eichler and Gottschalk (33) focus on relative differences in nor’easter frequency and intensity between El Niño and La Niña events, but they do not report absolute storm numbers or long-term trends. Hirsch et al. (34) develop a nor’easter climatology for 1950–1997, applying a cyclone algorithm to long-term reanalysis data, examining variations in frequency, minimum SLP, and other variables. They identify a marginally significant increase over time in storm minimum pressure, implying a slight weakening of nor’easters over time. While that observation would seem to run counter to theoretical expectations, it is SLP relative to the large-scale background, i.e., gradients in pressure rather than pressure itself, which are most closely tied to wind speed and therefore intensity (trends in the former may thus be influenced by changes over time in the large-scale background state rather than the storm characteristics themselves). Hirsch et al. do not assess trends in wind speed, a more direct measure of storm intensity. Colle et al. (20) estimate the number of nor’easters using the Climate Forecast System Reanalysis (CSFR) data, but their analysis is confined to a rather short period of time (1979–2004) and is dominated by interannual variability.

Another significant limitation in past observational studies is that they have tended to focus only on central tendencies rather than extremes. Elsner et al. (35) have shown that increases in hurricane intensity are most readily seen in the subset of the strongest storms, while Garner et al. (36) find that increases in storm surge are most pronounced in the upper quantiles of the probability distribution. It is thus of particular interest to investigate how nor’easter intensity is changing across the quantiles of the underlying statistical distribution.

Such is the purpose of the current study. We assess historical changes in nor’easters by applying a cyclone tracking algorithm to the ERA5 reanalysis dataset, providing a long-term historical record of nor’easter tracks and intensities extending from 1940 to 2025. We use maximum sustained wind speed as the measure of storm intensity, following the convention used for tropical storms (i.e., the Saffir–Simpson wind scale), and we use quantile regression to examine trends across the statistical distribution of intensity. We also assess changes in precipitation characteristics of the resulting nor’easter dataset.

## Tracking Nor’easters in the ERA5 Reanalysis

**Fig. 1.**
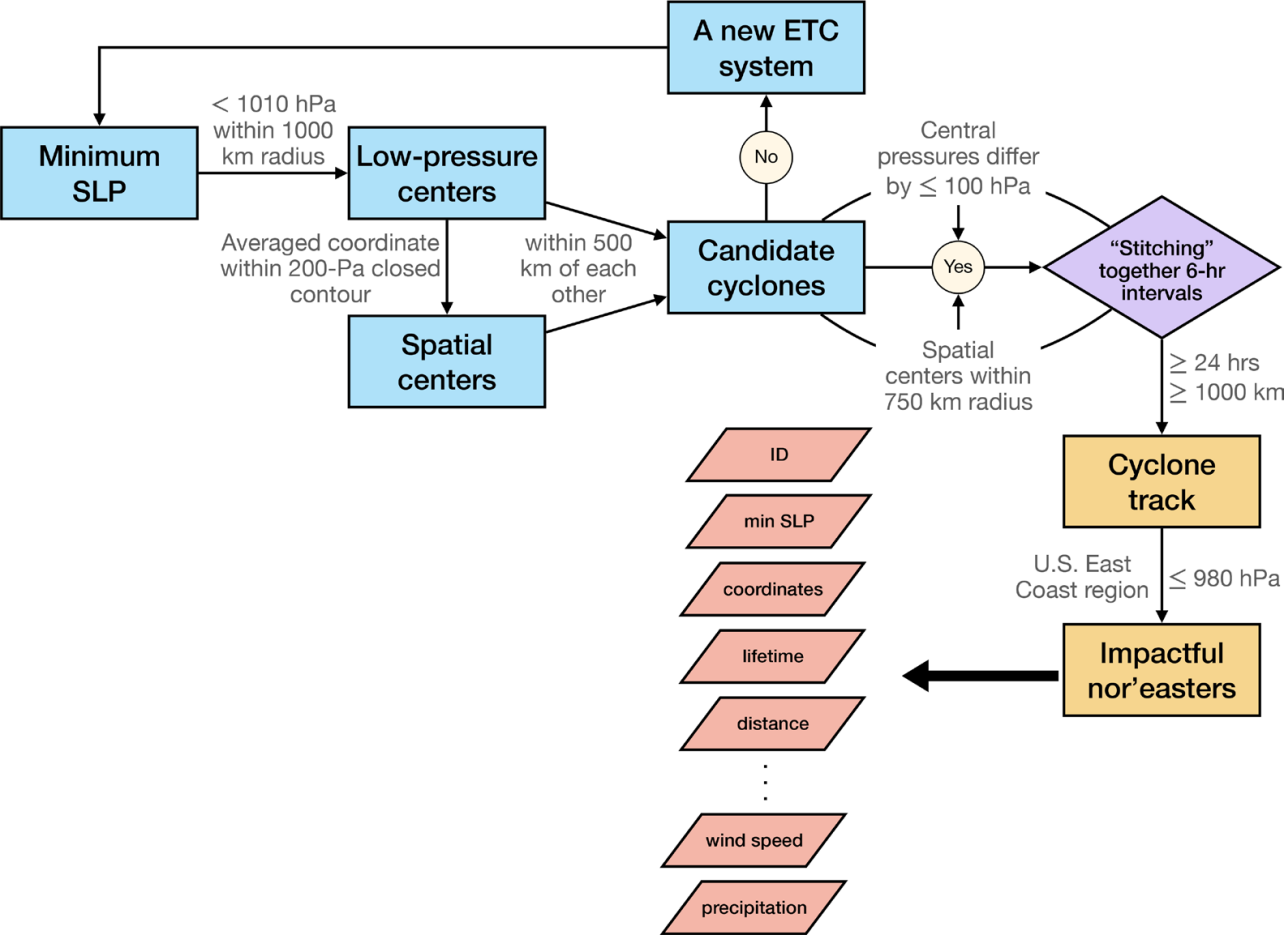
Workflow for nor’easter identification and tracking based on a two-step procedure. Step 1 involves identifying candidate cyclones at individual time steps (blue blocks). Step 2 constructs storm trajectories over time (purple and orange blocks). Key output variables used to define nor’easter characteristics are indicated by red diamonds.

**Fig. 2.**
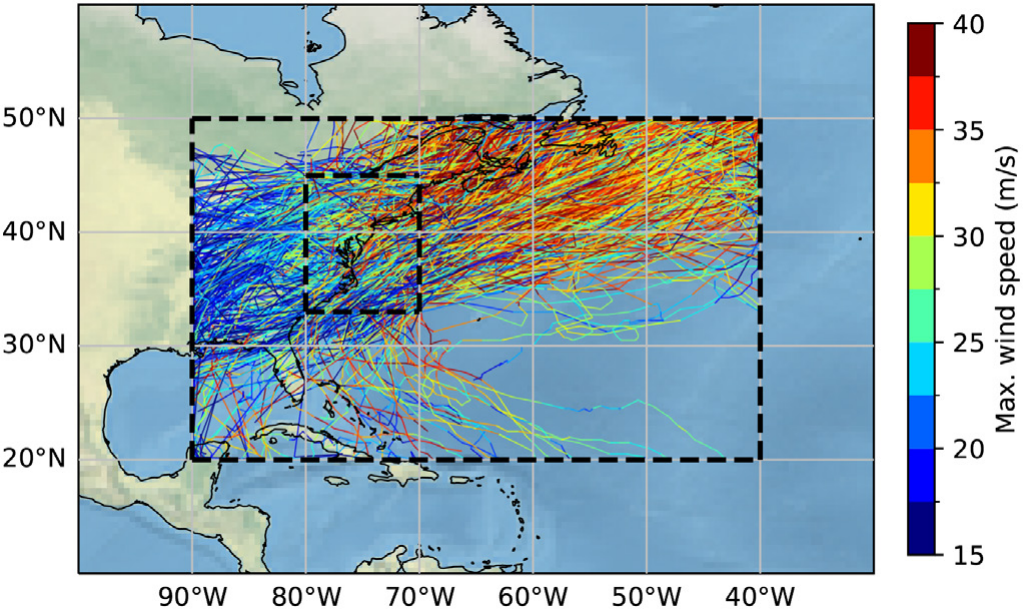
Nor’easter tracks for the period 1940–2025. Color scale represents intensities of tracks. The large dotted box corresponds to the domain where the tracking algorithm is applied (20°N to 50°N, 90°W to 40°W), while the smaller dotted box corresponds to the U.S. East Coast region (33°N to 45°N, 80°W to 70°W).

To identify and track the evolution of nor’easters, we utilize a Lagrangian cyclone tracking technique adapted from Michaelis et al. (4) and Bauer & Del Genio (1), using minima in mean SLP as the primary criterion (see Fig. 1 and also *Nor’easter Tracking Algorithm*). To filter out spurious ETCs and focus on relatively impactful nor’easters, we apply an objective definition of nor’easters such that an ETC must 1) travel a minimum distance of 1,000 km (5, 11, 20, 37) and persist for at least 24 h (1, 4, 20, 38), both of which are commonly used thresholds in ETC tracking; and 2) reach a minimum SLP of 980 hPa (consistent with the central pressure of a Category 2 tropical cyclone), ensuring the exclusion of low-intensity nor’easters with limited destructive potential. Since our main focus is on storm tracks along the U.S. East Coast (33°N to 45°N, 80°W to 70°W; Fig. 2), we include only those tracks that intersect this region for at least one time step. The identified nor’easter tracks are further verified against documented storms from previous literature (8, 39) and news articles, with a comprehensive list of 108 nor’easters provided in *SI Appendix*, Table S1.

By applying the tracking algorithm to ERA5 reanalysis data from 1940 to 2025, we identify a total of 900 nor’easters, equating to an average of 10.6 per year. The trajectories and intensities—defined by the maximum 10-m wind speed within a 750-km effective storm radius of the storm center—of all identified nor’easters are illustrated in Fig. 2. Notably, nearly all (94%) historically documented nor’easters in the time range of the data are successfully captured in the reanalysis dataset.

**Fig. 3.**
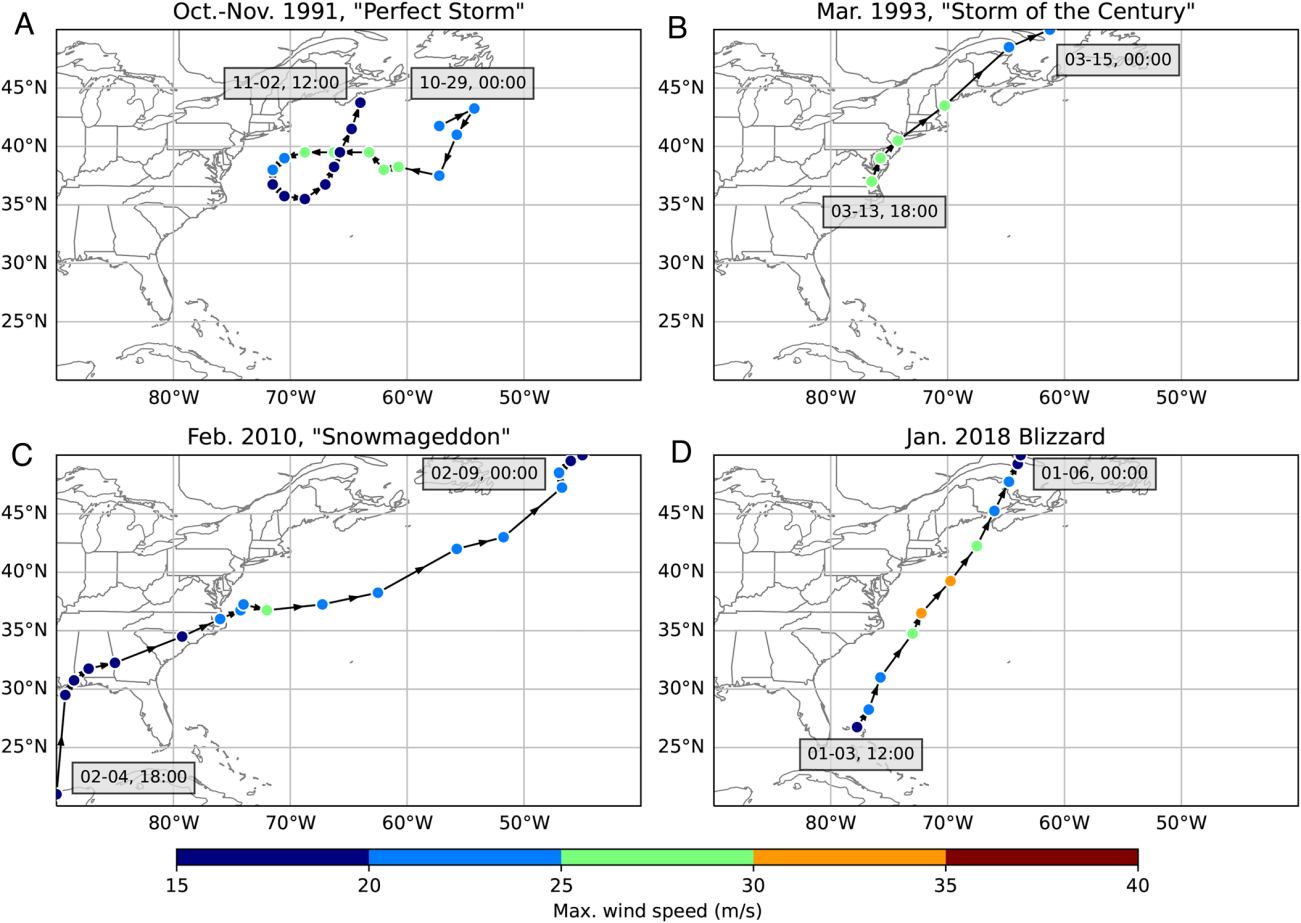
Map of four notable nor’easters. (*A*) Perfect Storm. (*B*) Storm of the Century. (*C*) Snowmaggedon. (*D*) January 2018 blizzard. Dots along the tracks indicate storm intensity at each 6-h time step, color-coded by the maximum 10-m wind speed. The initial and final tracking times (MM-DD, HH) are also displayed.

The characteristics (including the minimum SLP and maximum 10-m wind) of the storms captured in our reanalysis dataset coincide closely with corresponding historical data. The trajectories of four notable historic storms are illustrated in Fig. 3. The Perfect Storm (Fig. 3*A*) was a nor’easter that merged with a hurricane (29), reaching a lifetime minimum SLP of 975.7 hPa and a maximum wind speed of 28.8 m/s. The Storm of the Century (Fig. 3*B*) was one of the deadliest nor’easters on record, claiming 208 lives (40). It reached a lifetime minimum SLP of 961.3 hPa and a maximum wind speed of 31.7 m/s. Snowmaggedon (Fig. 3*C*) in 2010 reached a lifetime minimum SLP of 970.1 hPa and a maximum wind speed of 26.8 m/s, leaving over 230,000 homes without power (31). The January 2018 blizzard (Fig. 3*D*) was a record-breaking bomb cyclone (32) that reached a lifetime minimum SLP of 952.8 hPa and a maximum wind speed of 31.9 m/s.

It is important to note that the use of a 980 hPa minimum pressure threshold may exclude some impactful nor’easters. One notable example is the “Great Appalachian Storm” of 1950. This storm produced wind gusts exceeding 100 mph in Newark NJ, Concord, NH, and Hartford, CT, and was highlighted by Kocin and Uccellini (39) for its particularly devastating combination of extreme atmospheric elements (41). It serves as a reminder that not all ETCs with severe impacts exhibit exceptionally low central pressure. Some derive their intensity from a strong pressure gradient between a rapidly advancing cyclone and a retreating anticyclone. It is also important to note, however, that the Great Appalachian Storm was meteorologically atypical for a nor’easter, featuring unusual southeasterly surface winds that led some to refer to it as a “southeaster” (42). Aside from such important exceptions, our criteria are seen to capture the most historically noteworthy nor’easters.

## Trends in Nor’easter Intensity

**Fig. 4.**
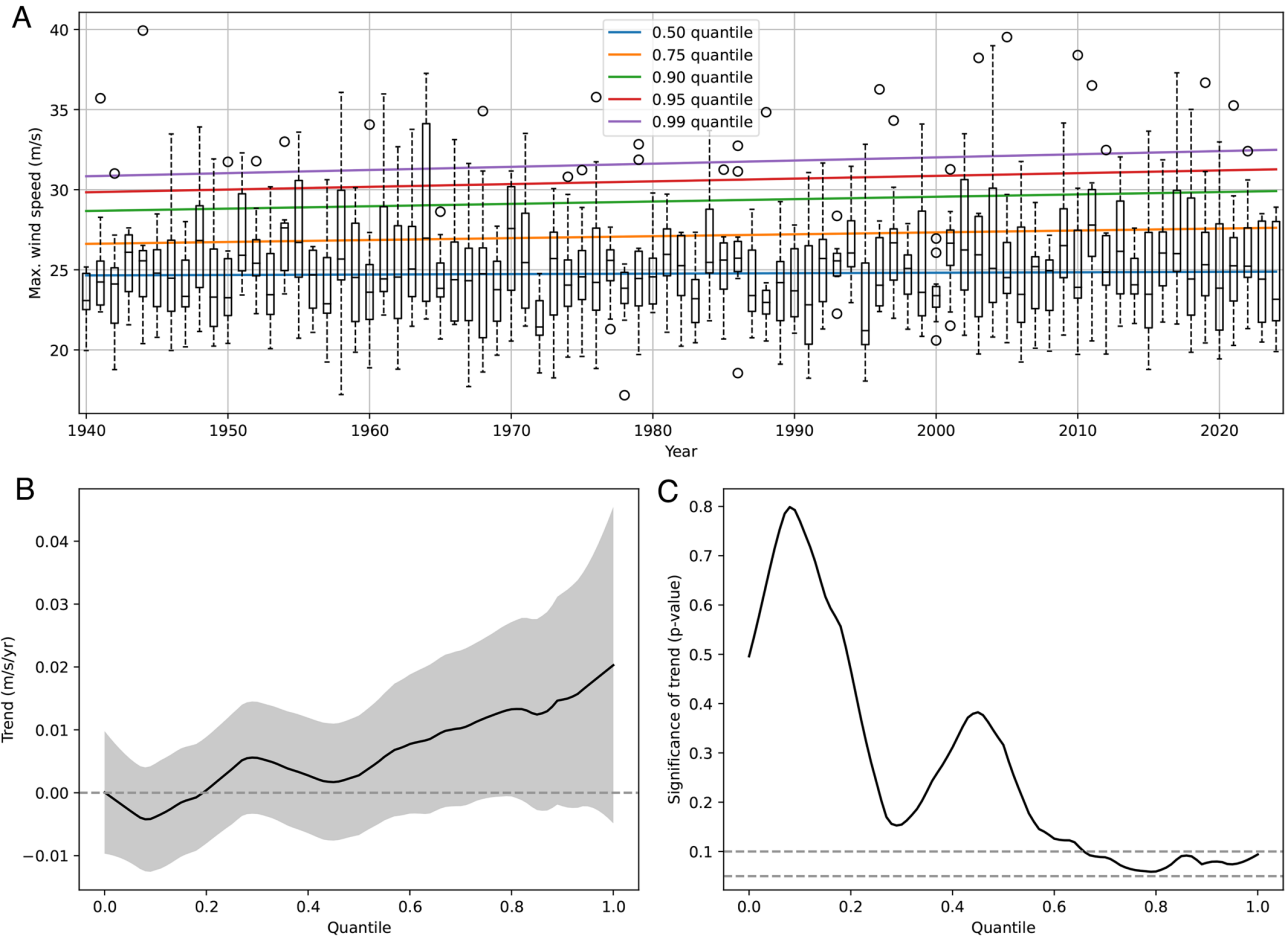
Trends in lifetime maximum wind speeds from 1940 to 2025. (*A*) Box plots of lifetime maximum wind speeds by year, with trend lines shown for five selected quantiles (median, 0.75, 0.90, 0.95, and 0.99). Whiskers extend up to 1.5 times the interquartile range, while open circles indicate values beyond this range. (*B*) Estimated trends across quantiles ranging from 0.01 to 0.99 in 0.01 intervals, with coefficients derived from quantile regression. The gray shading denotes the 90% CI, assuming independent and identically distributed errors. (*C*) Statistical significance of the trend at each quantile, determined using least-squares regression of wind speed as a function of year. One-sided *P*-values are reported based on a Wald test, where the null hypothesis is that the slope of the linear regression is not greater than zero. Dotted lines denote significance levels of *P* = 0.10 and *P* = 0.05.

To investigate how the intensities of nor’easters have changed over time, we employ a quantile regression approach, following the methodology of Elsner et al. (35). Unlike ordinary least-squares regression, quantile regression allows for trend analysis across different conditional quantiles of lifetime maximum wind speeds of nor’easters (35) (*Quantile Regression*). We specifically analyze the median and four upper quantiles (0.75, 0.90, 0.95, and 0.99), as shown in Fig. 4*A*. While there is no significant trend in the median lifetime maximum wind speed, the trend becomes increasingly pronounced at higher quantiles, suggesting that the most intense nor’easters are strengthening over time.

To evaluate the significance of trends, we use both linear least-squares regression (Fig. 4 *B* and *C*) and the nonparametric Mann–Kendall test (43) (*Mann–Kendall Trend Analysis*), allowing us to assess the robustness of the observed trends and estimated statistical significance. Notably, the largest trends are observed for the higher quantiles. Trends, for the least squares quantile regression, become statistically significant at *P* < 0.10 for quantiles above 0.66. A similarly pronounced increasing trend at higher quantiles is also evident when applying the Mann–Kendall trend analysis (*SI Appendix*, Fig. S1 *A* and *B*).

The precise significance levels vary depending on the choice of statistical test, time interval, and effective storm radius. Trends from the Mann–Kendall analysis, for example, are statistically significant at *P* < 0.10 for quantiles 0.58 to 0.97, and at *P* < 0.05 for quantiles 0.62 to 0.85 (*SI Appendix*, Fig. S1*C*). Of specific potential concern is the sensitivity of the trend to changes in input data sources during the transition from traditional surface and radiosonde observations in the early part of the record to multisensor observations in later years. However, we find that the trends of interest (*SI Appendix*, Fig. S2) are even greater in magnitude and of equal or greater statistical significance if confined entirely to the satellite era (1979–2025), with *P* < 0.10 for quantiles 0.57 to 1.0 and *P* < 0.05 for quantiles 0.6 to 0.78 or >0.95. While the exact details of the trend analysis vary somewhat based on the timeframe analyzed (e.g., 1950–2025; *SI Appendix*, Figs. S3 and S4) and the effective storm radius used (e.g., 500 and 1,000 km; *SI Appendix*, Figs. S5–S8), the results overall lead to a clear finding: the strongest nor’easters are becoming stronger.

## Trends in Nor’easter Precipitation

To investigate the precipitation rates associated with nor’easters, we calculate the mean hourly precipitation by dividing the total precipitation volume of a storm by its lifetime (*Nor’easter-Related Wind Speed and Precipitation Extraction*). The total precipitation is determined by integrating the depth of water equivalent over a 750 km effective storm radius (42), or a 1,500 × 1,500 km^2^ grid centered on the storm’s low-pressure center. To account for the sensitivity to the selected radius, 500 km (4, 8) and 1,000 km are also considered (*SI Appendix*, Fig. S9).

**Fig. 5.**
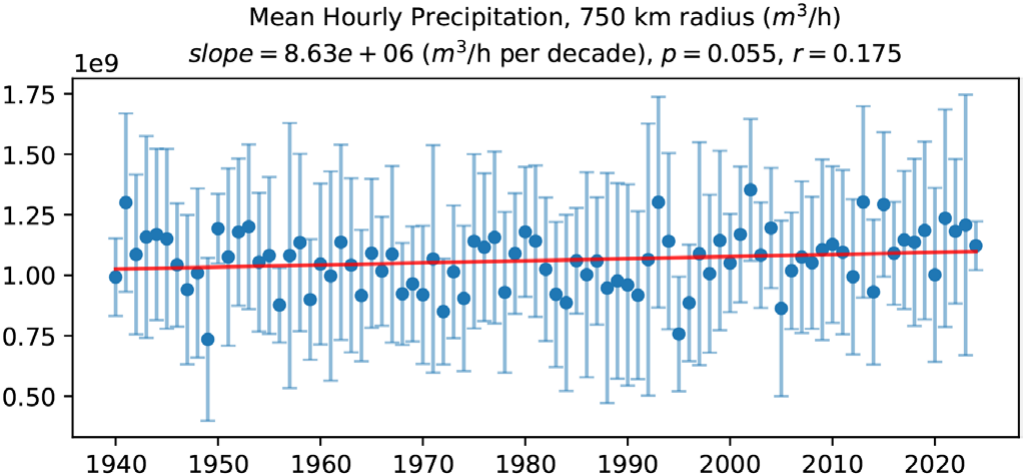
Time series of hourly precipitation of nor’easters and associated annual mean trends over the period 1940–2025. The blue circles represent the annual mean hourly precipitation (m^3^/h), while the error bars indicate one SD. The red line indicates the linear trend. The values of *P* and *r* denote the *P*-value from a hypothesis test (with the null hypothesis that the slope is zero) and the Pearson correlation coefficient, respectively.

Fig. 5 shows the time series of mean hourly precipitation across the nor’easters in our dataset. There is an increasing trend in mean hourly precipitation for an effective storm radius of 750 km (*P* = 0.055) with warming over the past eight decades. The trend is more significant at larger effective storm radii (*SI Appendix*, Fig. S9), with a statistically significant increase at the *P* < 0.05 level for 1,000 km (*P* = 0.034). This increase in precipitation rates is expected from the Clausius–Clapeyron relation since a warmer atmosphere has a greater capacity for moisture.

## Discussion

In summary, we have shown that the strongest nor’easters have intensified over the past century, a trend consistent with model projections indicating an increase in more intense ETCs along the U.S. East Coast. This intensification, as discussed earlier, is expected due to increased storm moisture, fueled by warmer ocean temperatures, leading both to increased latent heating, and greater coastal baroclinic instability. We also observe an increasing trend in nor’easter precipitation rates, consistent with stronger storms that are associated with heavier snowfall accumulation. Our observational study complements recent studies using the high-resolution regional climate model simulations (4, 8, 12) pointing toward an intensification of nor’easters on a warmer planet.

Our findings have direct implications for managing coastal hazards associated with the prospect of stronger nor’easters. Flood risk associated with tropical cyclones has already risen significantly for east coast cities such as New York City (44). The potential additional contribution to coastal flooding risk from intensified nor’easters has not been accounted for in past such assessments. The famous Ash Wednesday storm in early March 1962—one of the more notorious nor’easters from the standpoint of coastal damage, caused widespread devastation along the U.S. East Coast. The total economic loss from this event was estimated at approximately $3 billion (1962 USD). When adjusted for inflation, a storm of similar magnitude striking today would result in losses exceeding $21 billion (2010 USD) (45). Accounting for inflation, that would be equivalent to $31 billion, which is in proportion to the typical cost of a major landfalling hurricane. Given the significant increase in coastal development in historically impacted areas, this estimate likely underrepresents the true potential economic consequences of a comparable event in the present day.

The prospect of stronger nor’easters may imply the counterintuitive possibility of increased winter cold air outbreaks in regions neighboring the U.S. East Coast, due to increased cold advection on the westward flank of intensified coastal cyclones, similar to what was seen in the mid-Atlantic region of the U.S. East Coast with the Jan 2018 blizzard. The potential for greater likelihood of future super-nor’easters, akin to the 1993 Storm of the Century and Feb 2010 Snowmaggedon, driven by a combination of intense convection, explosive cyclogenesis, portends prospects of paralyzing snowfalls, dangerous storm surges, and episodic cold extremes, underscoring the urgent need for coordinated efforts to assess and mitigate the devastating impacts of future such storms.

## Materials and Methods

### ERA5 Data.

The ERA5 global reanalysis dataset, produced by the Copernicus Climate Change Service of the European Centre for Medium-Range Weather Forecasts (ECMWF), provides hourly estimates of climate data from 1940 to the present (46). Unlike observational data, which can be regionally inconsistent, reanalysis integrates observations into numerical weather models to generate a spatial and temporal complete dataset with a horizontal resolution of 0.25° × 0.25° (~31 km). For this study, we use the “ERA5 hourly data on single levels from 1940 to present” dataset from Hersbach et al. (47), obtained from the Copernicus Climate Change Service website. The data were downloaded in hourly increments for the nor’easter storm season (September to April). Our analysis primarily focuses on 1940 to 2025. As the pre-1950 data are subject to greater uncertainties, because of, e.g., the absence of upper air observations before the mid-1940s (48), we also consider the 1950–2025 timeframe to assess the robustness of our results. The spatial domain of this dataset spans 20°N to 50°N and 90°W to 40°W, which encompasses the primary U.S. East Coast region influenced by nor’easters.

### Nor’easter Tracking Algorithm.

The cyclone tracking algorithm of Bauer and Del Genio (1) and Michaelis et al. (4) is adapted here for identifying and tracking ETCs, including nor’easters. This algorithm employs a Lagrangian tracking technique, which allows for the analysis of individual storm systems over time (4). Among the most commonly used parameters for storm tracking are mean SLP and 850-hPa relative vorticity (1, 4, 5, 8, 11, 20, 37, 38). Mean SLP-based tracking methods are generally biased toward slower-moving systems, while vorticity-based approaches tend to produce more spurious storms (1). Consistent with Bauer and Del Genio and Michaelis et al., our analysis uses mean SLP as the primary parameter for storm identification and tracking, following a two-step procedure (see also Fig. 1):

First, identification of candidate cyclones at a single time step. The tracking algorithm identifies ETCs as minima in the SLP field. Specifically, it scans the spatial domain for grid points with SLP values below 1,010 hPa that are also local minima within a 1,000 km radius (equivalent to a 2,000 × 2,000 km^2^ grid). These grid points are designated as the low-pressure centers of candidate cyclones. For each identified low-pressure center, a 200-Pa closed contour is constructed using a flood-fill recursive algorithm. This contour captures the spatial features of the candidate cyclone, providing more detailed information than a single grid point. The average location of all grid points within the closed contour, weighted by the inverse of SLP, is considered the spatial center of the candidate cyclone. To ensure a coherent structure, candidate cyclones are retained only if the low-pressure and spatial centers lie within 500 km of each other, filtering out candidate cyclones without a well-defined center. The tracking algorithm operates at 6-h intervals, consistent with the standard time step in prior ETC tracking studies (1, 4, 5, 11, 20, 37, 38). An example of this identification process is provided in *SI Appendix*, Fig. S10, which shows a clear low-pressure system (blue shading) in the mean SLP field at a single time step. The algorithm identifies the lowest pressure point and constructs a surrounding 200-Pa closed contour (white). The low-pressure center is marked with a red dot. This process is repeated for each time step.

Second, construction of storm trajectories over time. To generate storm tracks, the algorithm links together candidate cyclones identified in consecutive time steps. A candidate cyclone at a given time step is paired with one from the previous step if their spatial centers lie within a search radius of 750 km—based on the assumption that ETCs do not exceed propagation speeds of 125 km/h (49)—and their central pressures differ by no more than 100 hPa. If a match cannot be found, the cyclone is considered the start of a new ETC system. To filter out spurious cyclones, additional criteria are applied, following established practices: Storm tracks are excluded if they persist for less than 24 h (1, 4, 20, 38) or travel less than 1,000 km (5, 11, 20, 37).

To focus our analysis specifically on nor’easters, we delineate the U.S. East Coast region with a bounding box of 33°N to 45°N and 80°W to 70°W, as shown in Fig. 2. This spatial domain is in line with that used in the University Corporation for Atmospheric Research (UCAR) Earth Observing Laboratory (EOL) nor’easter project (50). Storm tracks that do not pass through this region for at least one time step are excluded from the analysis. In order to exclude low-intensity nor’easters with limited destructive potential, we also require that a storm reaches a minimum SLP of 980 hPa over the course of its lifetime.

### Nor’easter-Related Wind Speed and Precipitation Extraction.

Wind speed and total precipitation are widely used indicators of storm intensity, as they are most directly related to the societal and economic impacts of nor’easters (11). Maximum wind speed (m/s) is defined as the highest 10-m wind speed recorded within the area of the nor’easter over its lifetime. We use an effective storm radius of 750 km to define the area of a nor’easter (equivalent to a 1,500 × 1,500 km^2^ grid around the low-pressure center). Past research has employed a variety of definitions for the area of ETCs. Studies on storm-relative compositing of ETCs have used ~500 km radius grids (4, 8). Booth et al. (51) and Nissen et al. (52) use 750 km and 1,200 km, respectively, as radii for ETC association (44, 53). We adopt an intermediate value of 750 km for our main analysis, but we also examine the sensitivity of our results to the precise choice. Additional analyses are performed with both 500 km (4, 8) and 1,000 km effective storm radii for wind speed (*SI Appendix*, Figs. S5–S8) and precipitation (*SI Appendix*, Fig. S9).

Hourly total precipitation (m) is obtained from ERA5 and represents the accumulated depth of liquid and frozen water—rain and snow—that reaches the Earth’s surface within each 1-h interval. The total volume of precipitation (m^3^) is calculated as follows:

[1] $$TVP=\sum_{t=t_{0}}^{t_{f}}\left(\sum_{i,j}w_{j}∙P∙A\right).$$

Here, *P* is the depth (m) of hourly precipitation over a grid cell, *A* is the physical area (m^2^) of a grid cell (0.25° × 0.25°) at the equator, and *w_j_* is a weight that adjusts for the latitude dependence of grid cell area. The volume of water equivalent is summed over each grid cell in a 750 km radius grid, where *i* and *j* are the longitude and latitude indices of each grid cell. This is summed over the storm’s lifetime (*t_0_* to *t_f_*) to obtain the total volume of precipitation (*TVP*). The mean hourly precipitation (m^3^/h) is then derived by dividing the total volume by the storm’s lifetime. While ETC-related precipitation is not commonly reported as a total volume, we have validated our results against the total snow volumes of nor’easters reported by Karvetski et al. (54) (*SI Appendix*, Supporting Text and Table S2).

While ERA5 data are available at hourly intervals, we run the tracking algorithm at 6-h intervals in accordance with past research on ETC tracking (1, 4, 5, 11, 20, 37, 38). Running the tracking algorithm at hourly intervals would also require significantly more computation time. However, analyzing storm-related quantities at 6-h intervals could lead to some uncertainties. For example, the true minimum lifetime SLP of a storm could have occurred between the 6-h intervals of the tracking algorithm. ERA5 data are available hourly, so it is possible to get more accurate results at a finer time resolution. Because the tracking algorithm operates at 6-h intervals, we do not have the exact coordinates of the storm centers at hourly time steps. We linearly interpolate five points between the storm center coordinates at 6-h intervals, approximate these points as the storm centers at each hour, and analyze certain variables (maximum wind speed, total precipitation) as described above. The linear interpolation to convert 6-h coordinates to hourly coordinates does assume that a storm moves in a relatively straight path between the 6-h coordinates. We consider this to be a reasonable approximation since analysis is done at relatively large effective storm radii. For the Perfect Storm of 1991, the minimum lifetime SLP values from ERA5 data are 977.2 hPa and 975.7 hPa for 6-h and 1-h intervals, respectively.

### Quantile Regression.

Quantiles are values obtained from the cumulative distribution function of a variable such that a certain portion of data points are at or below that quantile. For example, the 0.5 quantile (or 50th percentile) corresponds to the median, where half of the data values are at or below this value. Quantile regression extends traditional regression analysis by conditional quantiles of the dependent variable (35). In this study, we apply quantile regression to investigate trends in the lifetime maximum wind speed of nor’easters across different quantiles of the distribution. To further assess the robustness of these trends, we also repeat the analysis using the nonparametric Mann–Kendall trend test in place of linear least-squares regression.

### Mann–Kendall Trend Analysis.

While linear least-squares regression assumes that errors are normally distributed, the Mann–Kendall trend test is a nonparametric method that does not require the time series data to follow a normal distribution (43). Since the time series of nor’easter lifetime maximum wind speed exhibits no significant autocorrelation, we use the original Mann–Kendall test (original_test function from the pyMannKendall package) to estimate trends and their statistical significance across quantiles, from 0.01 to 0.99 quantile, in increments of 0.01. The original Mann–Kendall test returns a two-sided *P*-value by default; however, because we specifically hypothesize that ETCs are getting stronger under global warming, we report one-sided *P*-values by halving the two-sided output.

## Supplementary Material

Appendix 01 (PDF)

Movie S1. Animation of the mean sea level pressure field for the “Perfect Storm” from October 29 to November 2, 1991. Animation begins 1 day before and ends 1 day after the storm duration.

Movie S2. Animation of the mean sea level pressure field for the “Storm of the Century” from March 13 to March 15, 1993. Animation begins 1 day before and ends 1 day after the storm duration.

Movie S3. Animation of the mean sea level pressure field for the “Snowmaggedon” storm from February 4 to February 9, 2010. Animation begins 1 day before and ends 1 day after the storm duration.

Movie S4. Animation of the mean sea level pressure field for the January 2018 blizzard from January 3 to January 6, 2018. Animation begins 1 day before and ends 1 day after the storm duration.

## Data Availability

Code data have been deposited online at https://github.com/mann-research/KC-PNAS. Previously published data were used for this work (47).
