# Supplementary material for: The intensification of the strongest nor’easters
Source: Proc Natl Acad Sci U S A. 2025 Jul 14;122(29):e2510029122. doi: 10.1073/pnas.2510029122 (PMC12305023; doi:10.1073/pnas.2510029122)
Supplement: Supplementary file 1 — Appendix 01 (PDF) [file pnas.2510029122.sapp.pdf]

PNAS

Supporting Information for
The intensification of the strongest nor'easters

Kevin Chen[1], Xueke Li[2], Mackenzie M. Weaver[2], Shannon A. Christiansen[2*], Annabelle L. Horton[2] and Michael E. Mann[2*]

*Corresponding author: Shannon A. Christiansen and Michael E. Mann.
Email: chshann@sas.upenn.edu or mmann00@sas.upenn.edu

This PDF file includes:

Supporting text
Figures S1 to S10
Tables S1 to S2
Legends for Movies S1 to S4
SI References

Other supporting materials for this manuscript include the following:

Movies S1 to S4

Supporting Information Text

Validation of total precipitation volume calculation. Karvetski et al. (1) does a statistical analysis of the snowstorms presented by Kocin & Uccellini (2), which we reference in our documented nor'easter list (Table S1). Kocin & Uccellini (2) report the areas that receive a certain depth of snow, and Karvetski et al. (1) use this to estimate the total snow volume of each storm. We compare these total snow volumes with the total precipitation volumes we have calculated from ERA5 data, as shown in Table S2. The values are within 1 order of magnitude, with an 8% difference on average. While the values in Karvetski et al. (1) are snow volume (not snow water equivalent), their values are also underestimated, since areas with snowfall under 4 inches are not counted. In addition, snowfall is only reported for land area, while our calculation of total precipitation volume is for all grid cells in the domain. With these factors taken into consideration, the total precipitation volumes calculated from ERA5 reanalysis data are comparable to the total snow volumes reported in Karvetski et al. (1).

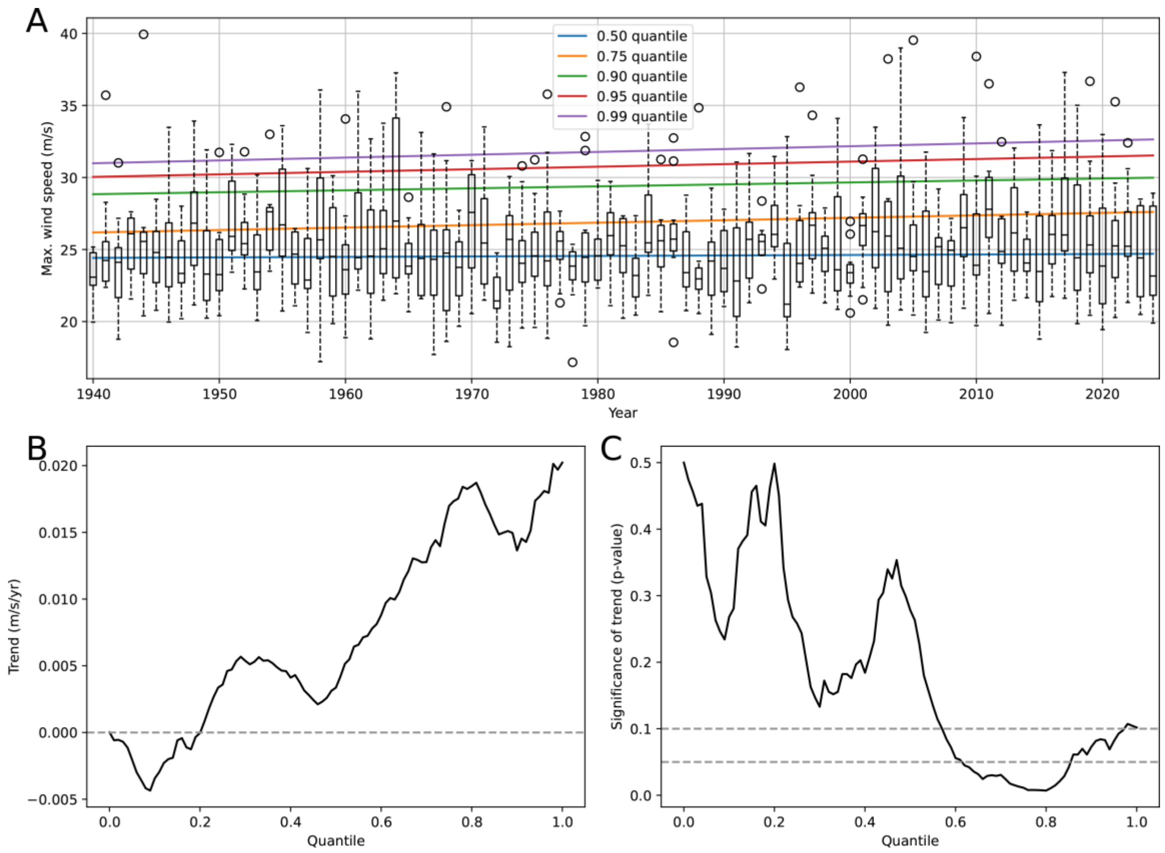

Fig. S1. Same as Fig. 4, but with trends and statistical significance estimated from the Mann-Kendall trend analysis. Trends are statistically significant at $p < 0.10$ for quantiles 0.58-0.97, and at $p < 0.05$ for quantiles 0.62-0.85.

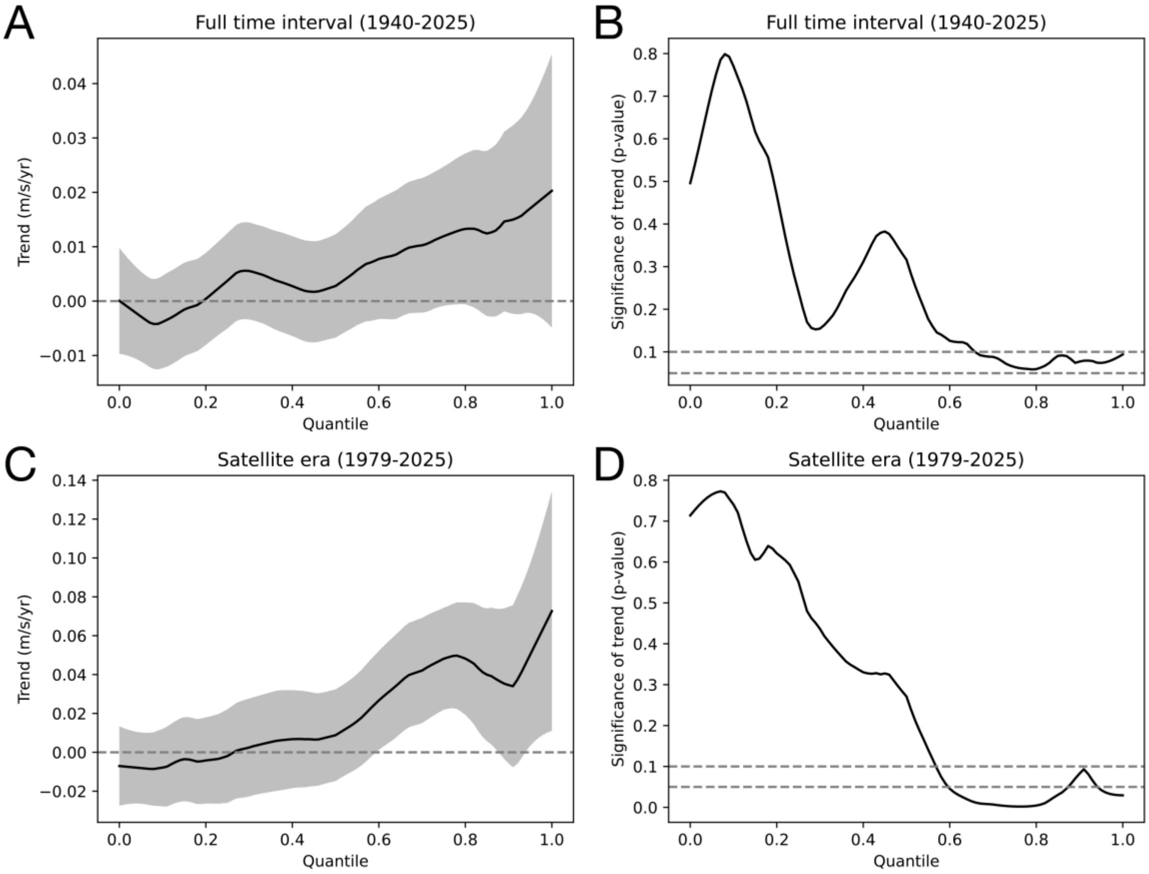

Fig. S2. Same as Fig. 4B and 4C, but for the full time interval (1940–2025) and the satellite era (1979–2025). Trends are statistically significant at $p < 0.10$ for quantiles 0.57-1.0 and $p < 0.05$ for quantiles 0.6-0.78 and >0.95.

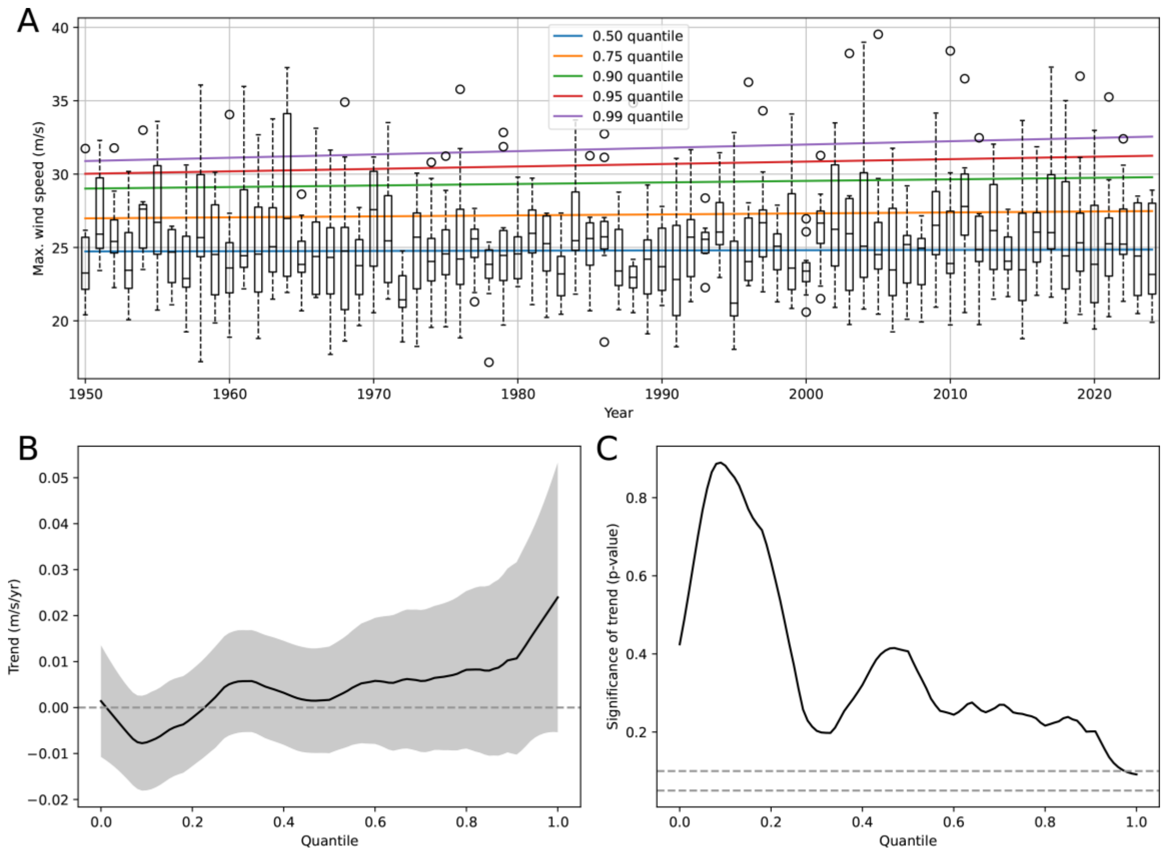

Fig. S3. Same as Fig. 4, but for the period 1950–2025. Trends are statistically significant at $p <$ 0.10 for 0.98-1.00.

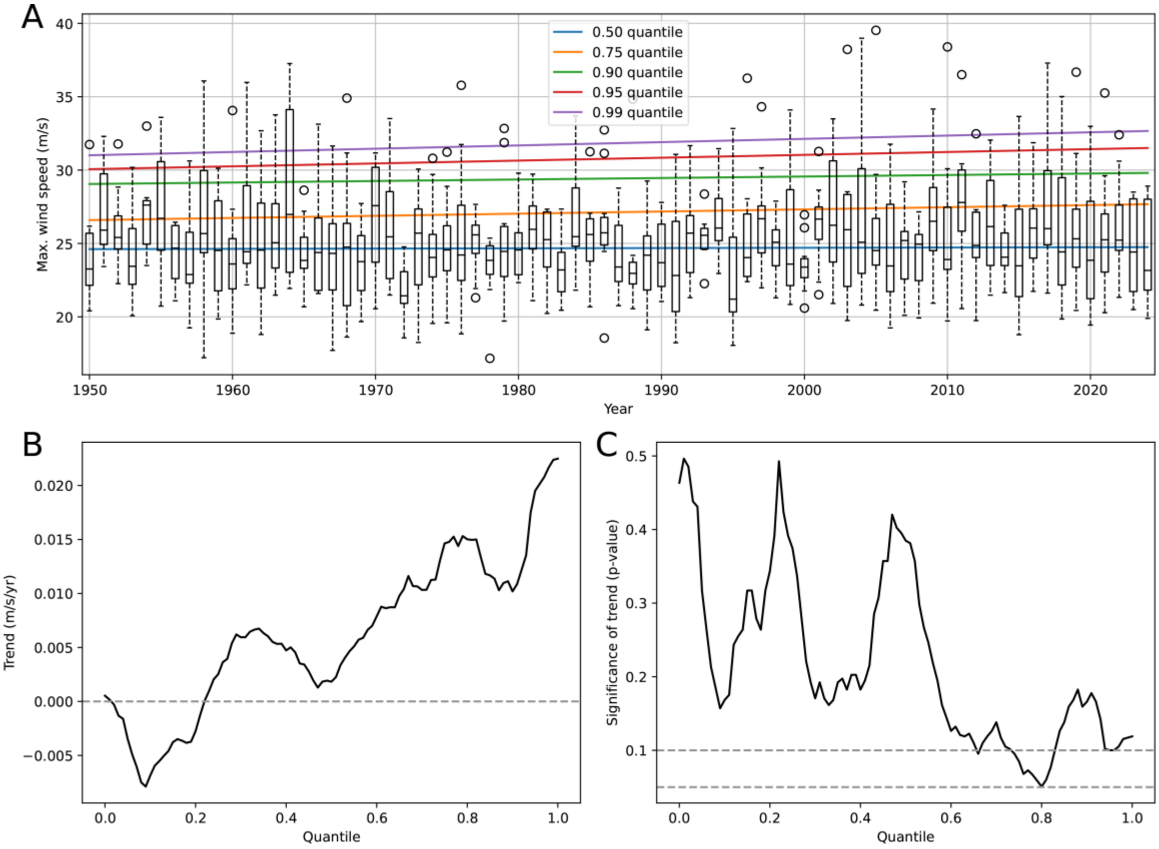

Fig. S4. Same as Fig. 4, but for the period 1950–2025, with trends and statistical significance estimated from the Mann-Kendall trend analysis. Trends are statistically significant at $p < 0.10$ for 0.66, and 0.74-0.82.

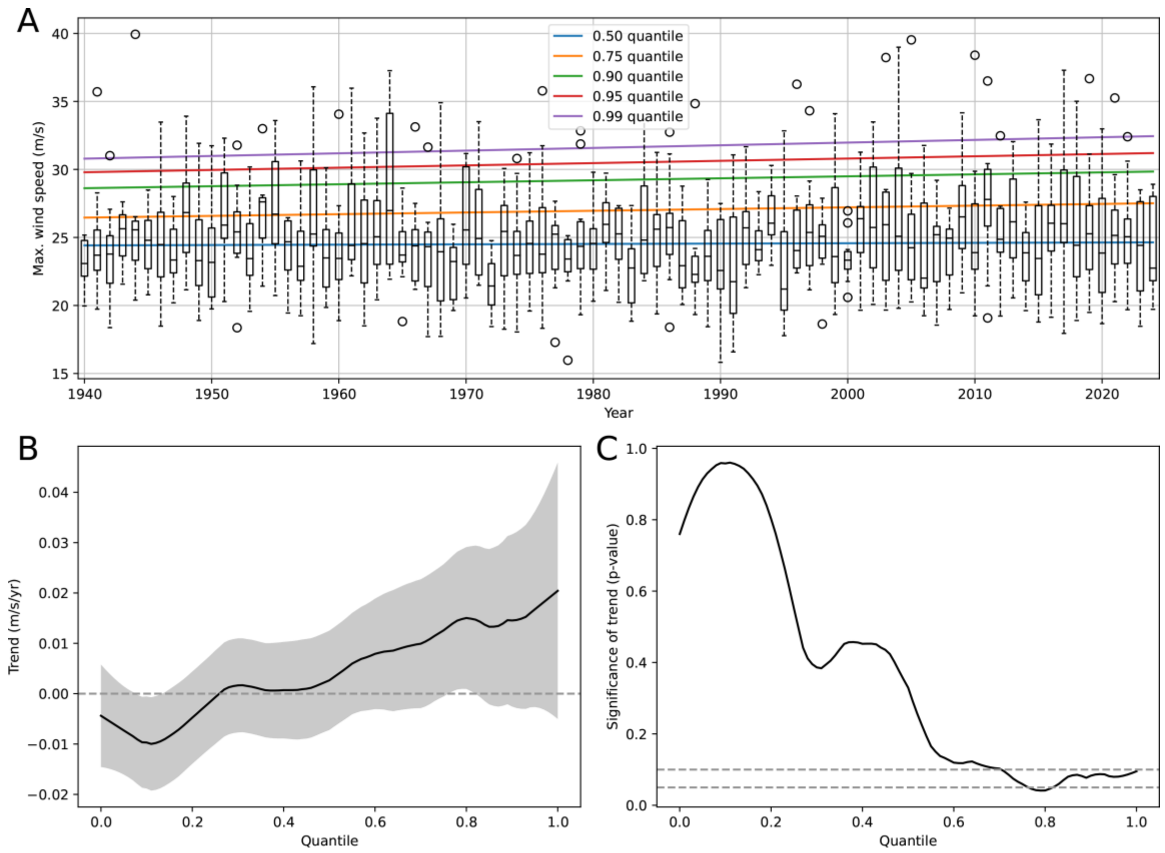

Fig. S5. Same as Fig. 4, but for an effective storm radius of 500 km. Trends are statistically significant at $p < 0.10$ for 0.71-1.00, and at $p < 0.05$ for quantiles 0.77-0.81.

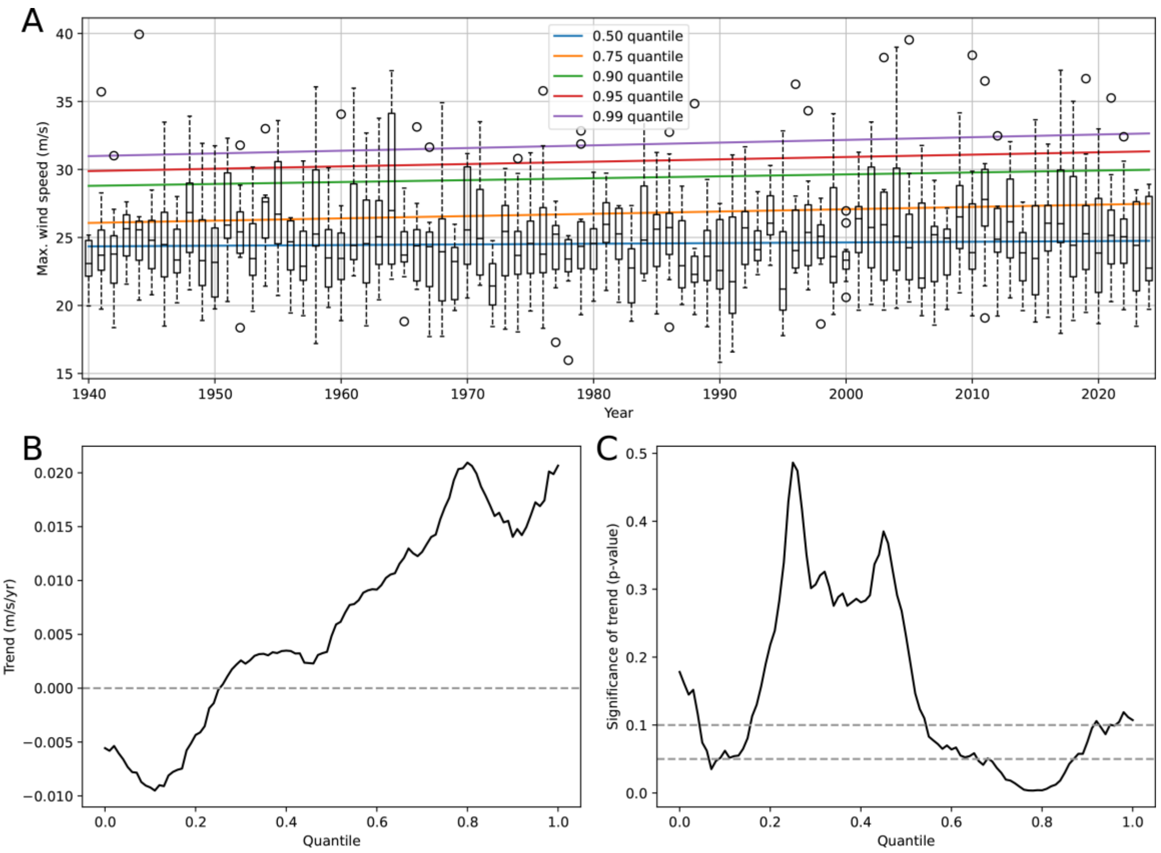

Fig. S6. Same as Fig. 4, but for an effective storm radius of 500 km, with trends and statistical significance estimated from the Mann-Kendall trend analysis. Trends are statistically significant at $p < 0.10$ for 0.05-0.15, 0.55-0.91, 0.93-0.94, and 0.96, and at $p < 0.05$ for quantiles 0.07-0.08, 0.66-0.67, and 0.69-0.86.

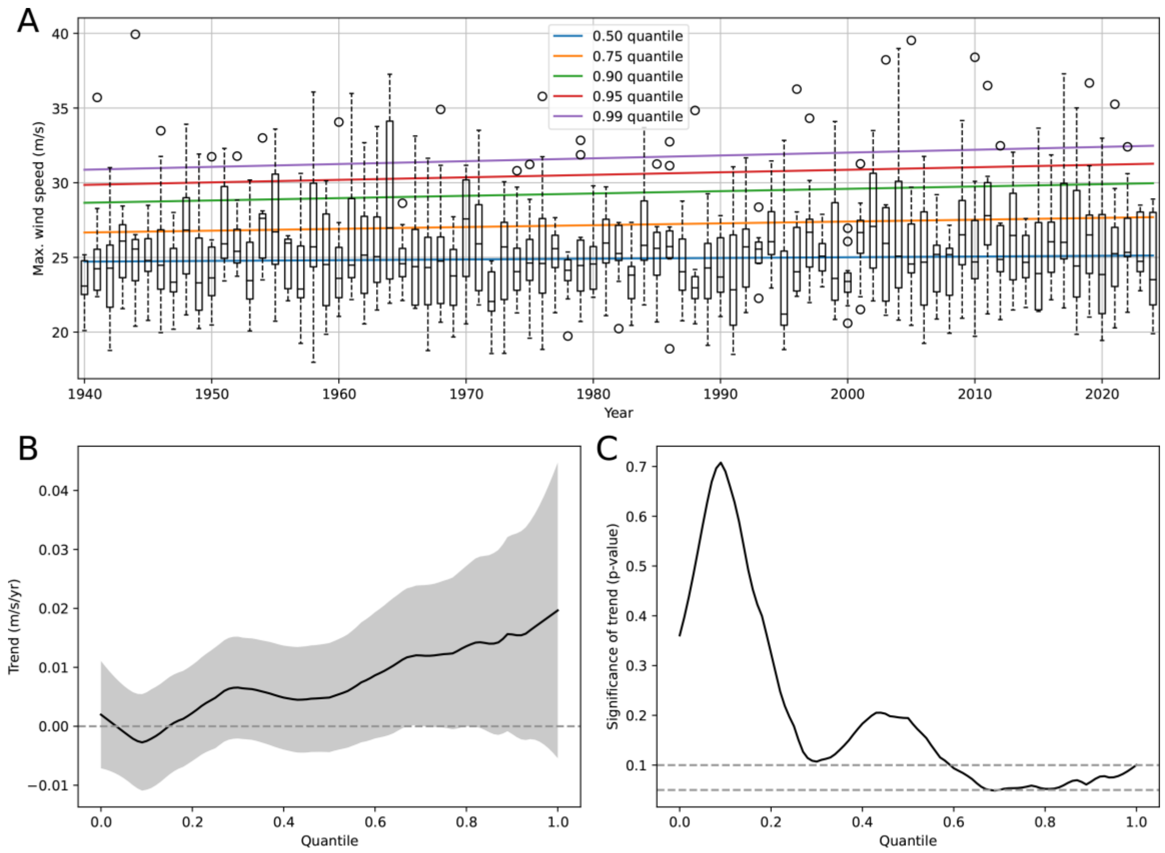

Fig. S7. Same as Fig. 4, but for an effective storm radius of 1000 km. Trends are statistically significant at $p < 0.10$ for 0.60-0.99, and at $p < 0.05$ for quantiles 0.68-0.69.

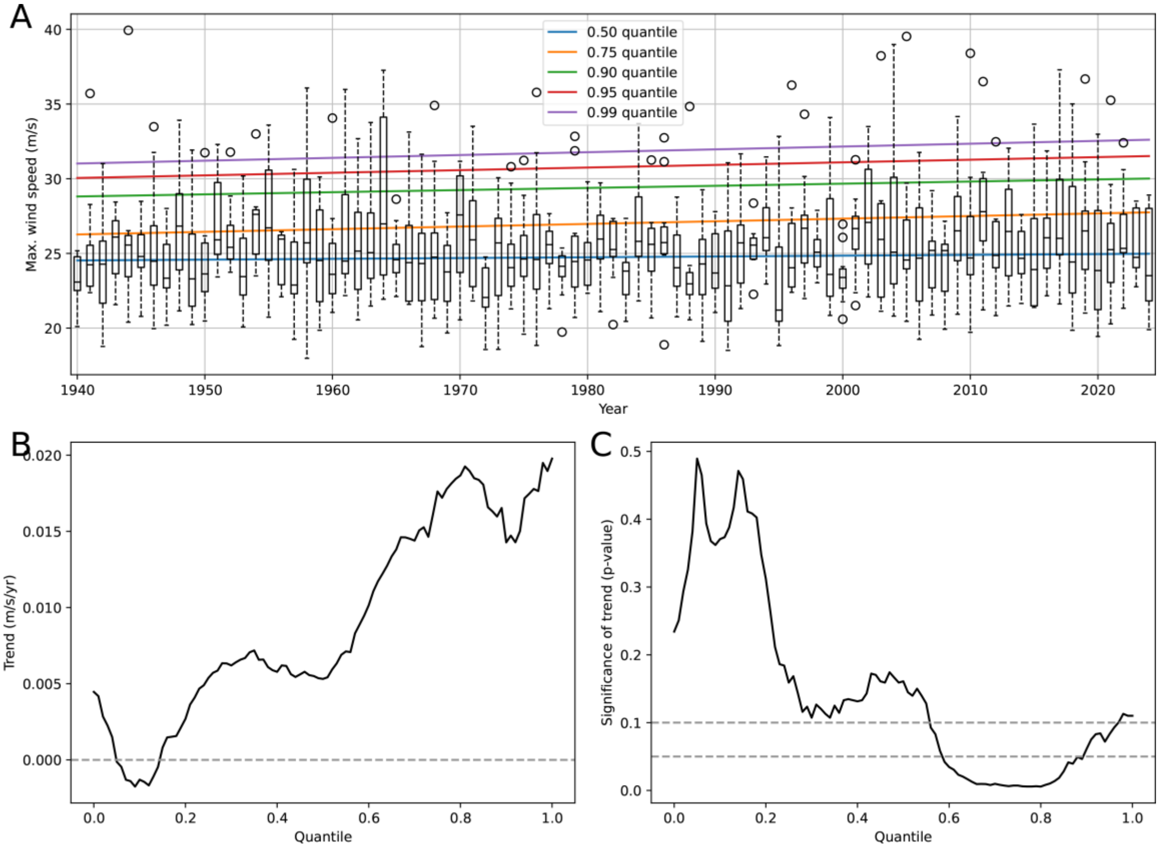

Fig. S8. Same as Fig. 4, but for an effective storm radius of 1000 km, with trends and statistical significance estimated from the Mann-Kendall trend analysis. Trends are statistically significant at $p < 0.10$ for 0.56-0.96, and at $p < 0.05$ for quantiles 0.59-0.89.

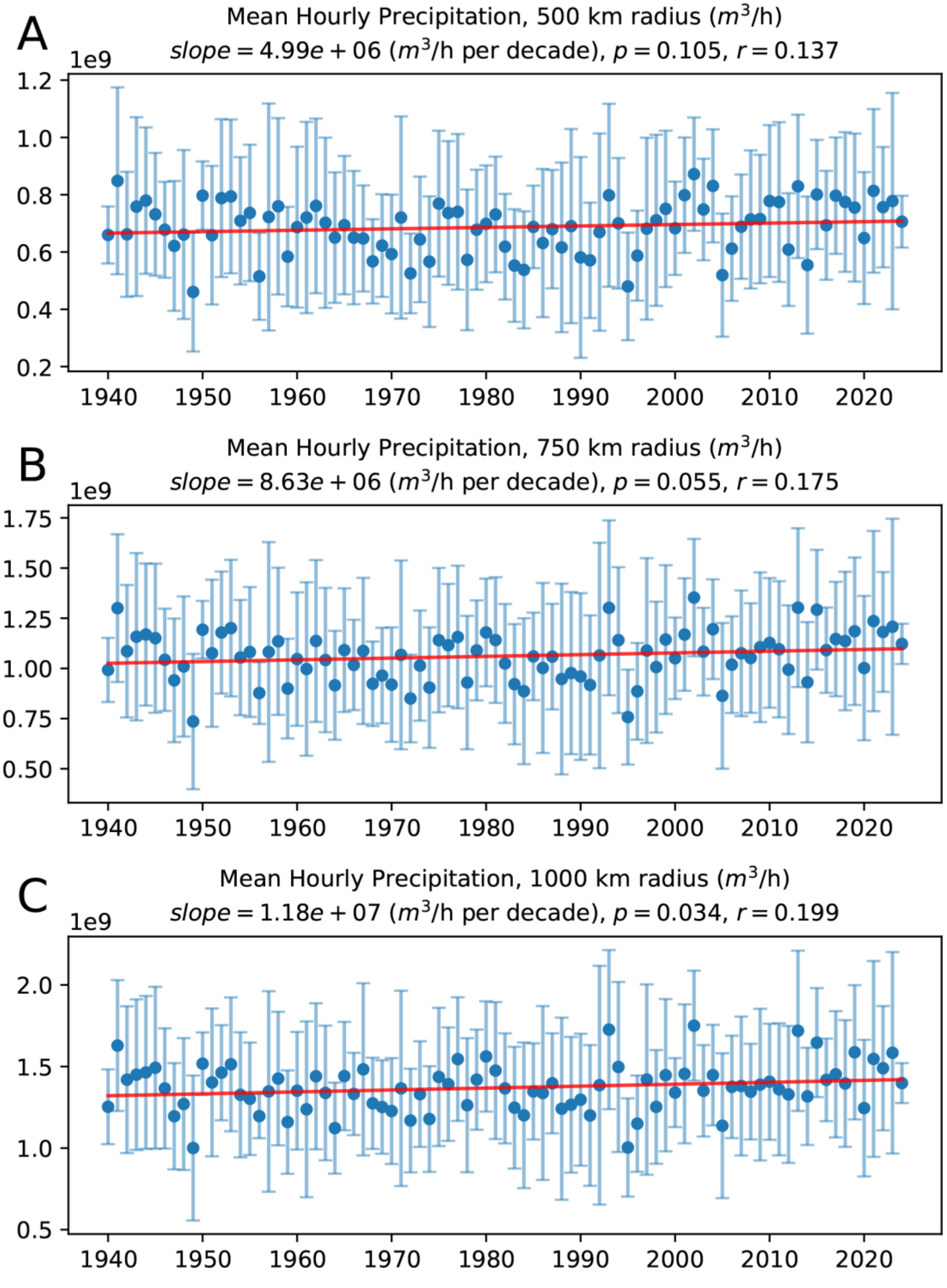

Fig. S9. Same as Fig. 5, but for the effective storm radii of 500 km, 750 km, and 1000 km.

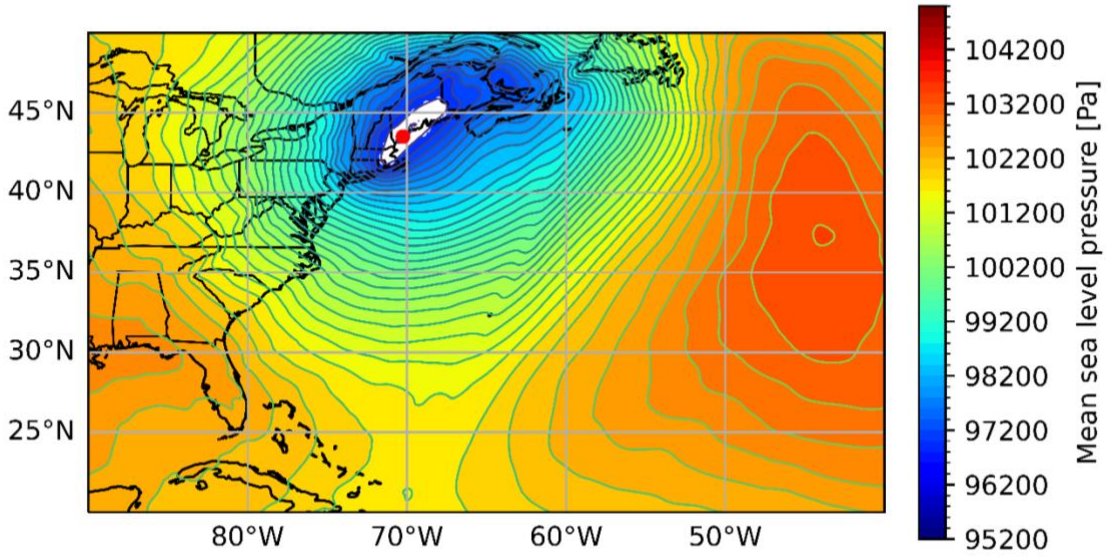

Fig. S10. Nor'easter tracking algorithm applied to a single time step for the mean sea level pressure field. The example shown is the "Storm of the Century" at 12:00 PM on March 14, 1993. Contour spacing is 200 Pa. The red dot indicates the low-pressure center of the storm. The 200-Pa closed contour around the low-pressure center is highlighted in white.

Table S1. Summary of 108 nor'easters compiled from Kocin & Uccellini (2), Marciano et al. (3), and news articles retrieved from the New York Times (NYT) archive before August 10, 2023. Minimum pressures are reported as tracked in the ERA5 reanalysis, with the exception of 6 storms not tracked by our algorithm. The last 3 columns indicate the source of the documented nor'easter.

Start (YYYY-MM-DD)	End (YYYY-MM-DD)	Minimum pressure from ERA5 (Pa)	Kocin & Uccellini (2004)	Marciano et al. (2015)	NYT
1952-02-17	1952-02-18	96952.8125	Yes	No	No
1953-11-06	1953-11-07	99349.5625	Yes	No	No
1956-01-07	1956-01-09	97614.875	Yes	No	No
1956-03-16	1956-03-17	97026.5625	Yes	No	No
1956-03-18	1956-03-20	100083.5	Yes	No	No
1957-12-04	1957-12-05	98110.4375	Yes	No	No
1958-02-14	1958-02-17	96830.375	Yes	No	No
1958-03-18	1958-03-21	97950	Yes	No	No
1959-03-12	1959-03-13	96450.5625	Yes	No	No
1960-02-13	1960-02-15	98067.5625	Yes	No	No
1960-03-02	1960-03-05	96477.4375	Yes	No	No
1960-12-10	1960-12-13	97341.8125	Yes	No	No
1961-01-19	1961-01-20	96870.25	Yes	No	No
1961-02-02	1961-02-05	97085.6875	Yes	No	No
1961-12-23	1961-12-25	97456.1875	Yes	No	No
1962-02-14	1962-02-15	99359.8125	Yes	No	No
1962-03-06	1962-03-07	96970.1875	Yes	No	No
1963-12-22	1963-12-24	N/A	Yes	No	No
1964-01-11	1964-01-14	98457.875	Yes	No	No
1964-02-18	1964-02-19	97604.375	Yes	No	No
1965-01-16	1965-01-17	99964.625	Yes	No	No
1966-01-22	1966-01-24	97654.9375	Yes	No	No
1966-01-29	1966-01-31	97171.0625	Yes	No	No
1966-12-23	1966-12-25	97635	Yes	No	No
1967-02-05	1967-02-07	99123.3125	Yes	No	No
1967-03-21	1967-03-22	99650.1875	Yes	No	No
1969-02-08	1969-02-10	96851.5	Yes	No	No
1969-02-22	1969-02-28	99010.25	Yes	No	No

1969-12-25	1969-12-28	97631.8125	Yes	No	No
1970-12-31	1971-01-01	98069.375	Yes	No	No
1971-03-03	1971-03-05	96043.375	Yes	No	No
1971-11-25	1971-11-28	99276.75	Yes	No	No
1972-02-18	1972-02-20	97329.4375	Yes	No	No
1973-12-16	1973-12-17	98567.5	Yes	No	No
1978-01-13	1978-01-14	99020.875	Yes	No	No
1978-01-16	1978-01-18	98905.5625	Yes	No	No
1978-01-19	1978-01-21	99024.9375	Yes	No	No
1978-02-05	1978-02-07	98331.6875	Yes	No	No
1979-02-18	1979-02-20	98064.0625	Yes	No	No
1979-10-10	1979-10-10	99720.375	Yes	No	No
1981-12-14	1981-12-17	97815	No	Yes	No
1982-01-13	1982-01-15	95174.5	Yes	No	No
1982-04-05	1982-04-07	96261.125	Yes	No	No
1983-02-10	1983-02-12	97516.25	Yes	No	No
1983-04-18	1983-04-19	98707.125	Yes	No	No
1984-03-08	1984-03-09	97894.8125	Yes	No	No
1984-03-28	1984-03-29	96608	Yes	No	No
1987-01-01	1987-01-02	98094.0625	Yes	No	No
1987-01-21	1987-01-24	96412.8125	Yes	Yes	No
1987-01-25	1987-01-27	98121.4375	Yes	No	No
1987-02-22	1987-02-24	94713.0625	Yes	No	No
1987-10-03	1987-10-04	98814.9375	Yes	No	No
1987-11-10	1987-11-12	96711.1875	Yes	No	No
1988-01-07	1988-01-08	98409.4375	Yes	No	No
1988-01-24	1988-01-27	96571.75	No	Yes	No
1990-12-27	1990-12-28	N/A	Yes	No	No
1991-10-29	1991-11-02	97572.8125	No	No	Yes
1992-01-02	1992-01-04	98239.1875	No	No	Yes
1992-09-26	N/A	N/A	No	No	Yes
1992-12-11	1992-12-12	98623.0625	Yes	No	Yes
1993-03-04	1993-03-08	97789.25	No	No	Yes

1993-03-12	1993-03-14	96132.375	Yes	No	No
1994-01-03	1994-01-06	97657.75	Yes	Yes	No
1994-01-07	1994-01-09	97214.5	Yes	No	No
1994-01-17	1994-01-17	N/A	Yes	No	No
1994-01-27	1994-01-28	98340.25	Yes	No	No
1994-02-08	1994-02-11	N/A	Yes	No	No
1994-03-02	1994-03-04	97161.375	Yes	No	No
1995-02-03	1995-02-05	96126.5625	Yes	No	No
1995-12-19	1995-12-21	95624.375	Yes	No	No
1996-01-06	1996-01-09	98434	Yes	Yes	No
1996-02-02	1996-02-04	100041	Yes	No	No
1996-02-15	1996-02-18	96948.5625	Yes	Yes	No
1996-04-09	1996-04-10	97460.5	Yes	No	No
1996-10-19	1996-10-20	99663.375	No	No	Yes
1997-03-31	1997-04-01	97872.5625	Yes	No	No
1998-02-22	1998-02-25	98253.625	No	Yes	No
1999-01-14	1999-01-15	98198.5625	Yes	No	No
1999-03-14	1999-03-15	97175.8125	Yes	No	No
2000-01-24	2000-01-26	97691.3125	Yes	No	No
2000-12-30	2000-12-31	98775.0625	Yes	No	No
2002-12-05	2002-12-07	97540.4375	No	No	Yes
2003-02-15	2003-02-18	100259.125	Yes	No	No
2003-12-05	2003-12-07	98613.1875	Yes	No	No
2006-02-10	2006-02-14	96644.5625	No	Yes	No
2009-02-28	2009-03-04	99989.3125	No	No	Yes
2009-12-18	2009-12-21	96666.25	No	Yes	No
2010-12-25	2010-12-28	96247.0625	No	Yes	No
2012-11-06	2012-11-09	98637.5	No	No	Yes
2013-02-07	2013-02-11	96968.9375	No	No	Yes
2014-01-03	2014-01-04	93536.125	No	No	Yes
2014-12-09	2014-12-15	99374.4375	No	No	Yes
2016-01-22	2016-01-24	98479.8125	No	No	Yes
2017-01-22	2017-01-27	96256.4375	No	No	Yes

2017-03-14	2017-03-17	97694.0625	No	No	Yes
2018-01-03	2018-01-06	95278.9375	No	No	Yes
2018-03-02	2018-03-09	97258.25	No	No	Yes
2018-03-07	2018-03-09	98575.6875	No	No	Yes
2018-03-12	2018-03-15	96932.9375	No	No	Yes
2018-03-19	2018-03-21	98872.9375	No	No	Yes
2020-03-23	N/A	N/A	No	No	Yes
2020-12-05	2020-12-08	97703.125	No	No	Yes
2020-12-16	2020-12-21	97400.375	No	No	Yes
2021-01-31	2021-02-04	98609.0625	No	No	Yes
2021-10-26	2021-11-04	97713	No	No	Yes
2022-01-29	2022-01-30	97044.4375	No	No	Yes
2022-12-16	2022-12-19	98781.6875	No	No	Yes
2023-03-13	2023-03-18	98208.8125	No	No	Yes

Table S2. Comparison of total snow volume from Karvetski et al. (1) and total precipitation volume from ERA5 for tracked storms that reach a minimum lifetime SLP of 980 hPa.

Start (YYYY-MM-DD)	End (YYYY-MM-DD)	Karvetski et al. total snow volume (m^3)	ERA5 total precipitation volume (m^3)
1956-03-16	1956-03-17	8.69E+10	6.01E+10
1958-03-18	1958-03-21	7.36E+10	6.95E+10
1959-03-12	1959-03-13	1.08E+11	8.94E+10
1960-03-02	1960-03-05	2.13E+11	9.53E+10
1960-12-10	1960-12-13	1.10E+11	1.22E+11
1961-01-19	1961-01-20	6.57E+10	3.94E+10
1961-02-02	1961-02-05	1.54E+11	9.51E+10
1961-12-23	1961-12-25	3.32E+10	7.12E+10
1962-03-06	1962-03-07	7.85E+10	1.36E+11
1963-12-22	1963-12-24	1.17E+11	6.01E+10
1964-02-18	1964-02-19	6.72E+10	8.11E+10
1966-01-22	1966-01-24	1.38E+11	1.51E+11
1966-01-29	1966-01-31	1.49E+11	9.00E+10
1966-12-23	1966-12-25	1.17E+11	6.36E+10
1969-02-08	1969-02-10	6.14E+10	9.21E+10
1969-12-25	1969-12-28	1.44E+11	1.46E+11
1971-03-03	1971-03-05	1.06E+11	8.57E+10
1972-02-18	1972-02-20	1.17E+11	5.88E+10
1982-01-13	1982-01-15	1.52E+11	8.26E+10
1982-04-05	1982-04-07	9.95E+10	8.04E+10
1983-02-10	1983-02-12	1.07E+11	1.32E+11
1984-03-08	1984-03-09	5.27E+10	4.30E+10
1984-03-28	1984-03-29	5.46E+10	1.29E+11
1987-01-21	1987-01-24	1.36E+11	9.32E+10
1987-02-22	1987-02-24	2.72E+10	1.17E+11
1993-03-12	1993-03-14	3.12E+11	6.42E+10
1994-03-02	1994-03-04	8.56E+10	1.68E+11
1995-02-03	1995-02-05	9.03E+10	6.87E+10
1995-12-19	1995-12-21	1.01E+11	1.58E+11
1996-02-15	1996-02-18	4.03E+10	7.19E+10

1997-03-31	1997-04-01	4.29E+10	8.53E+10
1999-03-14	1999-03-15	7.07E+10	1.22E+11
2000-01-24	2000-01-26	8.26E+10	9.51E+10

Movie S1. Animation of the mean sea level pressure field for the “Perfect Storm” from October 29 to November 2, 1991. Animation begins 1 day before and ends 1 day after the storm duration.

Movie S2. Animation of the mean sea level pressure field for the “Storm of the Century” from March 13 to March 15, 1993. Animation begins 1 day before and ends 1 day after the storm duration.

Movie S3. Animation of the mean sea level pressure field for the "Snowmaggedon" storm from February 4 to February 9, 2010. Animation begins 1 day before and ends 1 day after the storm duration.

Movie S4. Animation of the mean sea level pressure field for the January 2018 blizzard from January 3 to January 6, 2018. Animation begins 1 day before and ends 1 day after the storm duration.

SI References

1. C. Karvetski, R. Lund, F. Parisi, A statistical study of extreme nor'easter snowstorms. *Involve* **2**, 341–350 (2009).
2. P. J. Kocin, L. W. Uccellini, A Snowfall Impact Scale Derived from Northeast Storm Snowfall Distributions. (2004). https://doi.org/10.1175/BAMS-85-2-177.
3. C. G. Marciano, G. M. Lackmann, W. A. Robinson, Changes in U.S. East Coast Cyclone Dynamics with Climate Change. *Journal of Climate* **28**, 468–484 (2015).
