# Supplementary figures and images for: The intensification of the strongest nor’easters
Source: Proc Natl Acad Sci U S A. 2025 Jul 14;122(29):e2510029122. doi: 10.1073/pnas.2510029122 (PMC12305023; doi:10.1073/pnas.2510029122)

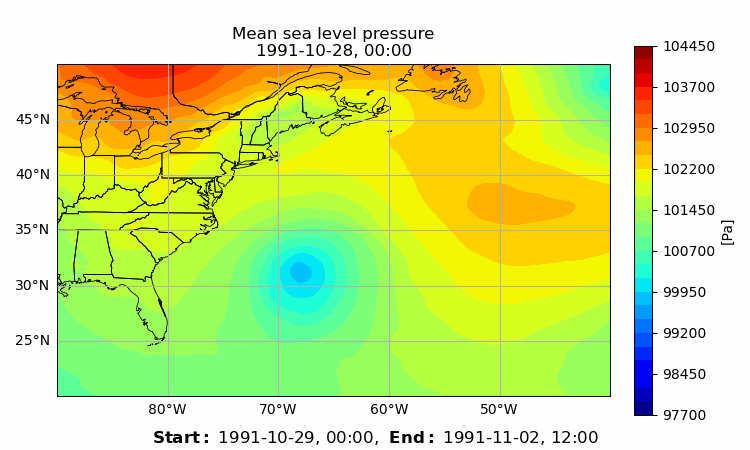

Supplement: Movie S1. — Animation of the mean sea level pressure field for the “Perfect Storm” from October 29 to November 2, 1991. Animation begins 1 day before and ends 1 day after the storm duration. [file pnas.2510029122.sm01.gif]

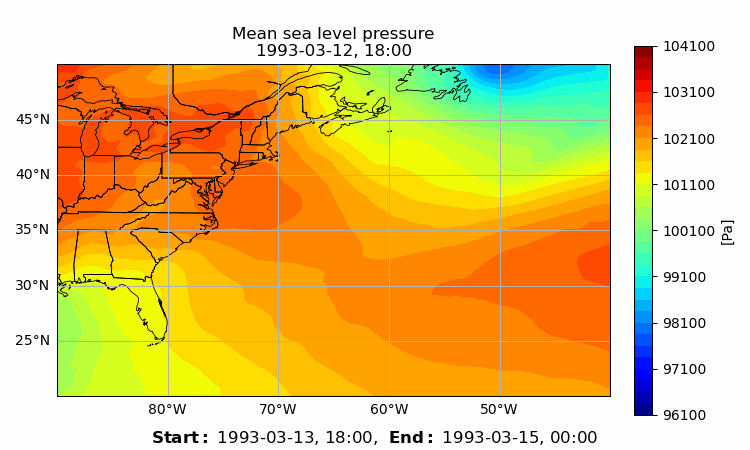

Supplement: Movie S2. — Animation of the mean sea level pressure field for the “Storm of the Century” from March 13 to March 15, 1993. Animation begins 1 day before and ends 1 day after the storm duration. [file pnas.2510029122.sm02.gif]

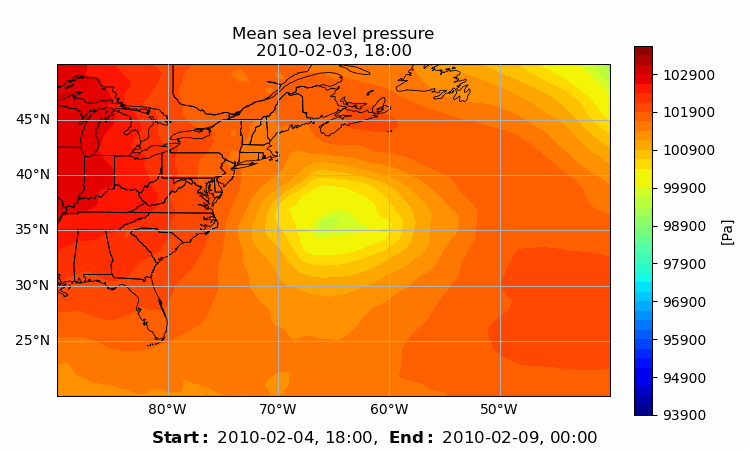

Supplement: Movie S3. — Animation of the mean sea level pressure field for the “Snowmaggedon” storm from February 4 to February 9, 2010. Animation begins 1 day before and ends 1 day after the storm duration. [file pnas.2510029122.sm03.gif]

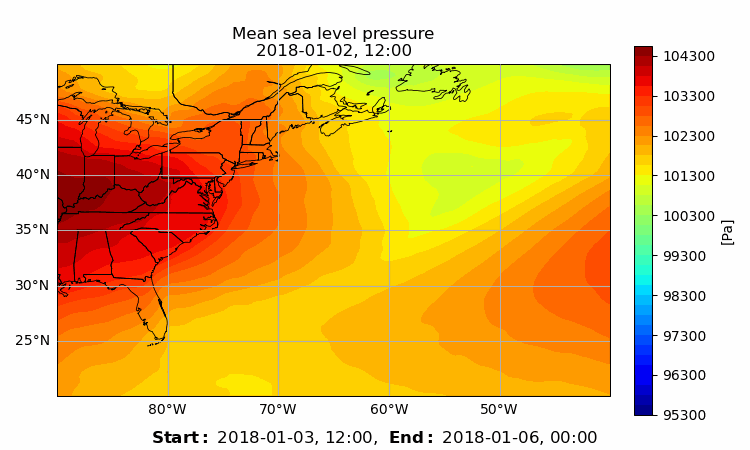

Supplement: Movie S4. — Animation of the mean sea level pressure field for the January 2018 blizzard from January 3 to January 6, 2018. Animation begins 1 day before and ends 1 day after the storm duration. [file pnas.2510029122.sm04.gif]
